# Editorial: Recent Breakthrough in Gluten Contamination

**DOI:** 10.3389/fnut.2021.795271

**Published:** 2021-12-23

**Authors:** Anil K. Verma, Girdhari M. Sharma, Francesco Valitutti

**Affiliations:** ^1^Celiac Disease Research Laboratory, Università Politecnica delle Marche, Ancona, Italy; ^2^Center for Food Safety and Applied Nutrition, Food and Drug Administration, College Park, MD, United States; ^3^Pediatric Unit, Department of Maternal and Child Health, Azienda Ospedaliera Universitaria San Giovanni di Dio e Ruggi d'Aragona, Salerno, Italy

**Keywords:** celiac disease, gluten contamination, gluten-free diet, gliadin, ELISA

## Introduction

Gluten contamination is a serious health issue for celiac disease (CD) patients. Exposure to a small amount of gluten (>10 mg/daily) can trigger an intense immunological reaction sufficient to restore clinical symptoms. Less than 20 mg/kg gluten in food is considered a safe amount of gluten as established by the regulatory authorities. However, substantial gluten contamination in commercial gluten-free products has been reported in recent years. It is extremely important to quantify accurately the amount of gluten in food products. Immunological techniques (i.e., antibody-based ELISA) are generally considered reliable methods to quantify gluten in food products. However, due to certain limitations, they do not often achieve the necessary accuracy, especially in the case of hydrolyzed and high heat-processed food samples. In the last decades, several non-immunological methods, such as DNA- and proteomics-based methods have been evaluated for gluten quantification in food products. Although these methods showed their efficiencies, due to some drawbacks, they are not regularly in use. Hence, gluten contamination, despite these efficient tools, remains a significant issue. There is certainly an unmet need to develop a reliable gluten quantification method with high accuracy and precision, especially in challenging food matrices. This Research Topic was aimed to provide comprehensive information about current approaches to accurately quantify gluten in food products and their biological proxy (i.e., urine from CD patients).

In this special issue a total of eight articles have been published (five pieces of original research, two brief research reports, and one opinion paper). The opinion paper by Scherf et al. was submitted on behalf of the Prolamin Working Group (PWG) as a statement on the final rule by the U.S. Food and Drug Administration (FDA) regarding gluten-free labeling for foods containing fermented or hydrolyzed ingredients. The rule acknowledged the absence of a scientifically valid analytical method to accurately quantify gluten in such food products, and thus the compliance with requirements for the use of gluten-free claims for these foods will be evaluated based on evidence that the food or ingredient used is gluten-free before fermentation or hydrolysis.

Gluten-free labeling is used on food packages to communicate the absence of gluten ingredients and show that any unintentional gluten in the food is below the threshold value. A survey of gluten content in foods labeled as gluten-free can be a helpful tool for risk assessment and improving the quality of life for CD and gluten-sensitive patients. In a brief research report, Calderón de la Barca et al. analyzed the cost comparison of gluten-free labeled foods from north-western Mexico with their equivalent counterpart, which may contain gluten. Further, the authors reported the findings from select gluten-free labeled foods analyzed for gluten content by ELISA, and immunoreactivity with CD patient IgA. Good manufacturing practices, including the use of dedicated or clean equipment, can significantly help in reducing the gluten cross-contact in foods. In the second brief report, Thompson et al. reported the gluten content in fries from different restaurants that did not contain gluten in their ingredients but were fried in shared fryers used to prepare other products containing wheat as a gluten source. With recent advancements in gluten detection methods, testing is no longer restricted to the laboratories but has reached the hands of consumers. This makes gluten detection convenient at the place of food consumption, such as at home and restaurants. However, the proficiency of the method and user may differ in laboratory *vs*. food consumption sites. In an original research, Marić and Scherf studied one such portable gluten sensor using food samples containing varying gluten content. The authors reported the performance of the sensor and discuss the variability in results from select samples when analyzed by different users.

In recent years, proteomics has been increasingly used for gluten detection as well as characterization. Three other original articles of this special issue used such analytical tools to assess gluten. The use of oats in the diet of celiac patients has been a continued topic of interest, partly due to the debated safe level of consumption. Gell et al. studied the variability among oat proteins from different varieties and various countries and developed an estimation method for ranking the avenin-epitope content, which may have an application in the selection of oat variety. Nye-Wood et al. used LC-MS to compare the protein profile of wheat flour containing markedly reduced allergenic gluten with traditional wheat flour. The authors report findings on changes in the amount of gliadin and glutenin specific proteins, and allergenic epitopes proportion in the novel wheat flour. Escobar-Correas et al. studied the proteome of various ryegrass cultivars using LC-MS to identify gluten-like peptides and a possible approach to distinguish ryegrass and wheat gluten.

Finally, in an original article, Costantino et al. encompass the role of telemedicine and urinary gluten peptides detection in assessing dietary compliance for CD patients during the COVID-19 pandemic. Sars-Cov2 pandemic has negatively affected national health systems worldwide and telemedicine has proven to be a reliable tool to deliver health care in certain situations, e.g., CD follow-up.

In summary, the articles in this special issue provide insights on gluten assessment to ensure safe food choices are available for gluten-intolerant consumers. The topics covered range from gluten measurement to its complex proteomic analysis using various analytical tools.

## Author Contributions

AV prepared a draft concept of the special issue. GS and FV supplemented and corrected the concept. AV, GS, and FV prepared the list of authors for the special issue manuscripts and were the main corresponding editors. All authors contributed to the article and approved the submitted version.

## Conflict of Interest

The authors declare that the research was conducted in the absence of any commercial or financial relationships that could be construed as a potential conflict of interest.

## Publisher's Note

All claims expressed in this article are solely those of the authors and do not necessarily represent those of their affiliated organizations, or those of the publisher, the editors and the reviewers. Any product that may be evaluated in this article, or claim that may be made by its manufacturer, is not guaranteed or endorsed by the publisher.

